# Tennessee

**Published:** 1899-12

**Authors:** Jos. Plaskett

**Affiliations:** Secretary


Secretary.

### TENNESSEE VETERINARY MEDICAL ASSOCIATION.

The Fourth Annual Meeting was held in Nashville on Wednesday, November 8, 1899. In the absence of President Fenimore the First Vice-President, Dr. Scott, occupied the chair and called the meeting to order. The attendance was good, about half of the qualified veterinarians of the State being present. The minutes of the last meeting were read and adopted.

The Committee on Legislation through its Chairman, Dr. Rayen, reported that nothing had been accomplished at the last meeting of the Legislature toward securing protective legislation for the qualified veterinarians of the State. The committee in charge had met with little encouragement, and it was evident that much time must elapse and much hard work be done before any results could be hoped for.

There were no new applications for membership, as no new veterinarians had moved into the State since the last meeting.

The Secretary read a letter from Dr. Fenimore, of Knoxville, who had been President of the Association during the last year, in which he tendered his resignation as a member of the Association. It was moved and carried that the resignation be accepted.

Before the reading of papers various matters pertaining to the veterinary profession throughout the State were discussed in an informal manner, and many points of interest and mutual benefit were thus brought out. Dr. Scott then read his paper on "Some Diseases of the Horse's Foot from a Shoeing Stand-point." He handled his subject in a capable manner, and his remarks were based on sound practical sense, aided by a wide experience derived from the management of a large shoeing forge. The paper was freely discussed, every member present taking part.

He was followed by Dr. Bird with a paper on "Sanitary Science and Prevention." Dr. Bird touched briefly on the prophylactic measures necessary in order to stop outbreaks of certain diseases, dealing specially with the vaccination of animals for the prevention of anthrax and black leg. The detection of tuberculosis and glanders was also commented on, and the entire subject was discussed in a manner that showed the essayist to be a progressive and scientific veterinarian.

A vote of thanks was tendered to Drs. Scott and Bird for their excellent papers. The election of officers for the ensuing year resulted as follows: President, Dr. Scott; First Vice-President, Dr. White; Second Vice-President, Dr. Blackburn. Dr. Plaskett was re-elected Secretary and Treasurer. The time and place of the next meeting were left to the Executive Committee.

There being no other business, the meeting adjourned.

JOS. PLASKETT, D.V.S.,
Secretary.

